# The top three unanswered questions in the management of open fractures

**DOI:** 10.1097/OI9.0000000000000072

**Published:** 2020-03-23

**Authors:** Yousif Atwan, Emil H. Schemitsch

**Affiliations:** Division of Orthopaedic Surgery, Western University, London, ON, Canada.

**Keywords:** fracture healing, infection, nonunion, open fractures

## Abstract

Despite improvements in treatment strategies and emphasis on preventative measures, the management of open fractures continues to be a challenging endeavor for orthopaedic surgeons. Deep infections, delayed healing, and nonunion continue to be problematic complications associated with these devastating injuries. There remain many unanswered clinical questions regarding the management of these injuries and how the various aspects of care can be further optimized. There continues to be a paucity of evidence regarding how infection can best be treated and prevented, how to reliability predict bone healing/nonunion, and how bone healing can be best augmented in the setting of open fractures and their potential nonunions. This review aims to assess the current literature on these top unanswered questions and discuss the gaps in evidence that may be filled with future studies.

## 1 Infection prevention

Open fractures are associated with significant bone and soft tissue trauma in the setting of significant contamination.^[1]^ This allows pathogens to invade the breached soft tissue site and adhere to nonviable tissue or implant surfaces.^[2]^ Host immune defences and antibiotics are unfortunately often compromised by biofilm formation.^[1,2]^ The problem is exacerbated by significant vascular disruption leading to a decreased local concentration of systemically delivered antibiotics.^[1]^ Therefore, the rate of infection after open fractures has been as high as 30%.^[3,4]^ Despite best medical and surgical practice including early intravenous antibiotics and adequate debridement, infections continue to be problematic and cause a significant patient, healthcare, and socioeconomic cost burden.^[3,4]^

With over 2 million fracture fixation implants utilized per year in the United States, implant-related infections remain a problem in current orthopaedic trauma practice.^[5,6]^ This challenging complication may lead to nonunion, delayed union, loss of function, or amputations in otherwise healthy patients.^[7,8]^ This has historically been a difficult complication to treat as the diagnosis has not always been clear. Without a clear international definition of fracture fixation-related infection, approaches to these presentations were often based on management principles of prosthetic joint infections.^[9]^ With the support of the AO Foundation, Metsemakers et al^[10]^ developed an international consensus on the definition, diagnosis, and management strategies for fracture-related infections.

Treatment of fracture-related infections depends on a variety of factors such as patient comorbidities, acuity of infection, causative organism, implant stability, stage of fracture healing, and soft tissue considerations.^[9,10]^*Staphylococcus aureus* and *Staphylococcus epidermidis* account for 70% to 90% of orthopaedic infections in the setting of fracture fixation or prosthetic joints.^[11]^*S aureus* and gram-negative bacilli are very virulent organisms that commonly cause early (<2 weeks) infections.^[9,12]^ At that stage, biofilm is often still at an immature phase and can usually be treated with irrigation and debridement, if the fracture fixation method remains stable and intact.^[9]^ Meanwhile, *S epidermidis* is a commensal organism of human skin that is usually harmless as it does not have the ability to penetrate skin on its own.^[13]^ Once it invades local tissue in the setting of open fractures, its low virulence allows it to remain minimally detected and develop a mature biofilm that is well suited to resist antibiotic therapy.^[9,14]^ Therefore, this bacterial biofilm formation has remained problematic in combatting fracture-related infections.

The Fluid Lavage of Open Wounds trial exhibited a 13% unplanned reoperation rate within 12 months of open fractures.^[15]^ This suggests that open fractures remain a significant problem despite advances in open fracture management. Moreover, many questions remain unanswered in the quest to prevent and eradicate fracture-related infections. It is generally accepted that the utilization of preoperative antiseptic solution does decrease the rate of surgical site infections.^[16]^ However, there is no clear evidence indicating the best antiseptic solution to use in the setting of open fractures.^[16,17]^ Large trials and surveys have demonstrated great variability in the practice patterns regarding antiseptic solutions utilized by orthopaedic trauma surgeons.^[15–17]^ Preoperative Aqueous Antiseptic Skin Solutions in Open Fractures is a current clinical trial evaluating if 10% povidone-iodine is more effective than 4% chlorhexidine at preventing surgical site infections and unplanned fracture-related reoperations. This trial aims to fill the void in evidence-based literature regarding preoperative antiseptic solutions in the setting of open fractures.

Disruption of vascular anatomy in the setting of open fractures, results in decreased local tissue concentration of intravenously delivered antibiotics.^[1]^ To bypass this deficiency, the use of local antibiotics at the wound site has become increasingly used. This provides greater local tissue concentrations of antibiotics compared with systemically delivered management.^[18]^ At a recent OTA meeting, a randomized control trial assessing the effect of locally delivered Vancomycin powder in the setting of open fractures on the rate of infection was presented.^[19]^ The study revealed that the rate of deep surgical site infection was 10.3% (95% CI 7.6–13.5) in the control group and 6.7% (95% CI 4.6–9.5) for the vancomycin treatment group with the resultant relative risk of 0.66 (95% CI 0.42–1.02; *P* = .07). Interestingly, post-hoc analysis of the deep infections found a rate of 7.8% for gram-positive bacteria within the control group and only 3.7% in the treatment group with relative risk of 0.48 (95% CI 0.27–0.85; *P* = .01). Meanwhile, gram-negative-only infections were 2.1% in the control group and 2.6% in the treatment group with relative risk of 1.25 (95% CI .54–2.91; *P* = .66). These findings correspond with known vancomycin activity against gram-positive organisms. Moving forward, the group has planned a secondary randomized control trial including a treatment group with vancomycin and tobramycin for full gram-positive and negative coverage. This will further delineate the role for local antibiotic delivery.

One delivery method historically used was antibiotic-loaded polymethylmethacrylate beads.^[20]^ Nonetheless, there has been considerable debate regarding this delivery technique. A study by van de Belt et al^[21]^ analyzed 6 different gentamicin loaded cement mixtures. Interestingly, they found that after 1 week, the antibiotic levels dropped to below detectable levels.^[21]^ Furthermore, only 4% to 17% of the incorporated antibiotic was actually eluted.^[21]^ McKee et al^[22]^ assessed 30 patients with long bone infections/infected nonunions and randomized them to antibiotic loaded polymethylmethacrylate vs antibiotic loaded bioabsorbable bone substitute. They found the use of bioabsorbable bone substitute had the potential to reduce the number of surgical procedures while maintaining a high rate of infection eradication.^[22]^ Nonetheless, this was a small study and required a larger scale trial. Level I evidence continues to be lacking in terms of the choice of ideal antibiotic, antibiotic dose, delivery substrate, and the timing of these interventions. A prospective clinical trial is currently underway assessing the efficacy of antibiotic impregnated calcium sulfate in the setting of infected tibial defects.

## 2 Predicting fracture healing

Despite advancing fracture care technology, overall fracture nonunion rates have been estimated to be 10% with rates as high as 33% for tibial shaft fractures postintramedullary nail fixation.^[23]^ Historically, there had been a lack of consensus on the definition of fracture nonunion as well as a method for assessing fracture healing.^[24]^ A 2002 survey of 444 orthopaedic surgeons revealed no consensus on the definitions of union and nonunion in the setting of tibial shaft fractures.^[24]^

Whelan et al^[25]^ assessed inter and intraobserver agreement of healing tibial fractures. They found significant interobserver agreement on number of cortices bridged by callus (κ = 0.75) and number of cortices with visible fracture line (κ = 0.7).^[25]^ With this information, they developed the Radiographic Union Scale for Tibial Fractures (RUST) score which ranges from 4 to 12 points based on assessment of 4 cortices and the presence of fracture lines and callus.^[25]^ RUST has been studied extensively assessing its reliability. A group of 7 reviewers assessed 45 diaphyseal tibial fractures and demonstrated significant intra and interobserver reliability with interclass correlation coefficients (ICC) of 0.88 and 0.86 respectively. With a larger sample size, Ali et al^[26]^ evaluated 345 tibial fracture radiographs by 2 reviewers at various time points and found an intraobserver ICC of 0.87 to 0.96 and interobserver ICC of 0.87 to 0.98. Furthermore, Tawonsawatruk et al^[27]^ demonstrated intraobserver ICC of 0.86 and interobserver ICC of 0.81 when 6 reviewers assessed 30 radiographs of rat tibial shaft fractures.

A modified RUST score was subsequently developed as it was argued that the RUST scoring system is dichotomous once healing has begun as once callus is present, it is scored as either having a present or absent fracture line. Some argued that since complete remodeling with loss of visible fracture line occurs late, further subdividing a RUST score of 2 (callus present with visible fracture line) to include either nonbridging or bridging callus would be beneficial.^[28]^ Therefore, the modified RUST score expanded the score to range from 4 to 16.^[28]^ In their initial manuscript, Litrenta et al^[28]^ indicated a slightly higher ICC for modified RUST (0.68) compared with that of RUST (0.63).

To validate these scores, biomechanical studies have been performed using plate and nail fixation models. Fiset et al^[29]^ assessed 29 adult rats with noncritical femoral shaft osteotomies repaired with a polyetheretherketone plate. They demonstrated great agreement with ICC of 0.89 and 0.86 for RUST and modified RUST respectively.^[29]^ Interestingly, it was noted that greater than 90% of contralateral femur load at failure was obtained by samples with RUST ≥ 10 and modified RUST ≥ 15.^[29]^ This suggested thresholds of “healed” plated fractures were a RUST score of 10 and modified RUST of 15. In an intramedullary nailing model, Litrenta et al^[30]^ suggested union was achieved at an average RUST of 10.4 and average modified RUST of 14.2.

A retrospective case-control study by Ross et al^[31]^ studied 323 patients with tibial shaft fractures and assessed risk factors for nonunion. Four out of the 40 collected variables were found to have statistically significant associations with nonunion. These variables were RUST, modified RUST, infection requiring intervention within 6 weeks and finally, the Non-union Risk Determination (NURD) Score.^[31]^ The NURD score is a nonunion prediction score that utilizes 5 points for flaps, 4 points for compartment syndrome, 3 points for chronic conditions, 2 points for open fractures, 1 point per class of American Society of Anesthesiologists Physical Status, 1 point for male gender and cortical contact.^[32]^ The chances of nonunion were 2% for NURD score from 0 to 5, 22% for NURD score from 6 to 8, 42% for NURD score 9 to 11, and 61% for a NURD score greater than 12.^[32]^

The study by Ross et al^[31]^ demonstrated that the NURD score was increasingly predictive of nonunion with decreasing RUST score. It was found that 25% of patients with a NURD score ≥7 and a RUST score between 6 and 9 went on to nonunion. In comparison, 69% of patients with a NURD score ≥7 and RUST score <6 experienced nonunion. Otherwise, all patients with RUST score ≥10 had fracture union regardless of NURD score.^[31]^ Overall, there has been tremendous advancement in the assessment of fracture healing and union. Further work is required to evaluate and simplify nonunion prediction in most long bone fractures.

## 3 Augmentation of fracture repair

Open fractures have a high propensity for infection, vascular compromise, and substantial bone loss, often occur in compromised hosts and these factors can subsequently impede fracture healing.^[33]^ Various grafting, bone substitution, or bioactive options have been utilized to enhance bone healing and the evidence surrounding their use is limited.^[33]^

Autologous bone grafting is the most common form of bone grafting and can be sourced from areas such as the iliac crest, distal femur, and proximal tibia.^[34]^ The iliac crest has historically been the most common site of such harvesting due to its ease of access and rich supply of progenitor cells.^[35]^ The use of iliac crest bone grafting in fracture nonunion has been long considered the gold standard option with union rates as high as 87% to 100%.^[36–40]^ Nonetheless, donor site morbidity remains a concern with this method and a void is left in search of noninvasive methods to augment fracture healing.^[41]^

Early methods utilized by orthopaedic surgeons to augment fracture healing included low-intensity, pulsed, ultrasound (LIPUS).^[42]^ The TRUST trial was a multicenter randomized control trial involving 501 patients to assess the effect of LIPUS on tibial shaft fractures treated with intramedullary nailing.^[43]^ Interestingly, those treated with LIPUS did not have improved clinical outcomes, faster return to function, earlier weight bearing, or accelerated radiographic healing parameters.^[43]^ Tarride et al^[44]^ completed a further analysis of this study and found no significant difference in health-related quality of life and determined that it was not a cost-effective method that should be utilized for this indication.

Bone morphogenic proteins (BMPs) are a group of proteins within the transforming growth factor beta family that have been shown to have a role in bone formation and healing through osteoinductive signaling.^[45]^ Multiple BMPs have been shown to be expressed during fracture healing within in vivo studies.^[46]^ The BMP Evaluation in Surgery for Tibial Trauma study was the first randomized controlled trial that attempted to assess the effect of BMP on the treatment of open tibial fractures.^[47]^ They demonstrated a 44% risk reduction of failure (defined as secondary intervention due to delayed union) in the BMP-2 treatment group compared with control.^[47]^ Despite significant results, controversy remained due to the trial being underpowered. A recent randomized controlled trial by Aro et al^[48]^ determined no significant difference in the healing of open tibial fractures with the use of BMP-2. A recent meta-analysis of evidence surrounding BMP use in fracture care demonstrated that for tibial nonunion, the use of BMP leads to similar results to that of autogenous bone grafting.^[49]^

In terms of cell-based therapy options, platelet-rich plasma (PRP) has been a widely-studied option in many orthopaedic presentations. A randomized controlled trial assessed the utilization of PRP and BMP-7 in the treatment of long-bone nonunions.^[50]^ The study included 120 patients and demonstrated 86.7% union rate in the BMP-7 treatment group compared with a 68.3% union rate in the PRP-treated group. A large meta-analysis demonstrated no evidence for efficacy of PRP in the setting of fracture healing or nonunion presentations.^[51]^

Bone marrow aspirate concentration (BMAC) has recently gained increased popularity as an alternative to iliac crest bone grafting.^[52]^ This utilizes a minimally invasive technique to harvest osteogenic mesenchymal stem cells in bone marrow.^[53–56]^ In an animal long bone model, the utilization of BMAC has demonstrated increased bone formation, higher torsional strength, and earlier bone healing when compared with control groups.^[56]^ Despite promising foundational studies, there remains a paucity of level I evidence regarding its utilization in open fractures and notably—nonunions. Furthermore, there are many protocols and methods utilized for BMAC harvesting and preparation and no consensus has been reached on the best method for its use.

## 4 Summary

Open fractures remain one of the most problematic presentations in orthopaedic trauma. They are at high risk for deep infections, delayed union, nonunion, and soft tissue compromise. Despite tremendous advances in fracture fixation, infection prophylaxis, and management plans, there remain several unanswered questions in the management of open fractures. Despite advances such as early intravenous antibiotic administration and meticulous surgical debridement, infection remains an issue and various local antibiotic delivery methods are lacking level I evidence. There have been advances in the use of radiographic scores to assess fracture healing but a gap remains in developing methods to predict nonunion at an early stage. Finally, numerous fracture-healing augments have been tested, but there is little level I evidence to support their use and nonunion remains a vital concern. This leaves many opportunities for future studies and trials to obtain level I evidence so that the knowledge gap in these vital aspects of open fracture management can be filled.
